# Efficacy of a 0.5% Propolis -0.9% Pomegranate Buccal Spray Treatment Compared with 2% Miconazole Gel for Denture Stomatitis Treatment in Elderly Patients: a Randomized Clinical Trial

**DOI:** 10.30476/DENTJODS.2021.91479.1588

**Published:** 2022-12

**Authors:** Yêda Maria Parro, Danielly de Mendonça Guimarães, Herick Sampaio Muller, Eduardo Barbosa Coelho, Andresa Aparecida Berretta, Jessica Aparecida de Lima, David De Jong, Vicen-te de Paulo Martins, Erica Negrini Lia

**Affiliations:** 1 Tropical Medicine Postgraduate Program, School of Medicine, University of Brasília, Brasília, DF, Brazil; 2 Dental Student, Dept. of Dentistry, School of Health Sciences, University of Brasília, Brasília, DF, Brazil; 3 Molecular Biology Postgraduate Program, Institute of Biological Sciences, University of Brasília, Brasília, DF, Brazil; 4 Dept. of Internal Medicine, Ribeirão Preto School of Medicine, University of São Paulo, Ribeirão Preto, SP, Brazil; 5 Apis Flora Industrial e Comercial Ltda, Ribeirão Preto, SP, Brazil; 6 Genetics Department, Ribeirão Preto School of Medicine, University of São Paulo, Ribeirão Preto, SP, Brazil; 7 Institute of Biological Sciences, University of Brasília, Brasília, DF, Brazil; 8 Dept. of Dentistry, School of Health Sciences, DF, Brazil

**Keywords:** Lythraceae, Denture stomatitis, Propolis, Clinical trial

## Abstract

**Statement of the Problem:**

Natural products have attracted interest as an alternative to synthetic medi-cations for the treatment of oral diseases due to their efficacy and safety. Propolis and pomegranate extracts have both demonstrated efficacy for the treatment of denture stomatitis. However, use of the two compounds together has not been tested for this purpose.

**Purpose:**

A comparison was made of the efficacy of a commercially available propolis-pomegranate buccal spray formulation for the treatment of denture stomatitis, compared with miconazole gel, based on stomatitis lesions and *Candida* spp. concentrations in mouth rinses.

**Materials and Method:**

This was an experimental study, characterized as an open-label, parallel two-armed, non-inferiority randomized clinical trial. Forty elderly adults aged < 60 years with denture stoma-titis were randomly allocated to two groups. The patients applied a buccal spray containing 0.5% propo-lis and 0.9% pomegranate extracts or 2% miconazole gel, a standard treatment recommended in Brazil, to the inner surface of their dentures three times a day for 14 days. They were examined at days 1, 7, 14 and stomatitis lesions were categorized according to Newton’s score. Mouth rinses were made with saline solution at days 1 and 14 and then assessed for *Candida* spp.

**Results:**

Both treatments reduced the Newton’s score, with clinical cure rates of 75 and 40% for the miconazole and propolis-pomegranate groups, respectively. The *Candida* concentrations in the mouth rinse decreased significantly only in the miconazole group.

**Conclusion:**

The propolis-pomegranate spray was less effective than the miconazole treatment. Howev-er, clinical improvement was also observed in patients treated with the propolis-pomegranate buccal spray.

## Introduction

Denture stomatitis (DS) is generally caused by *Candida* spp. [ 1 - 2 ]; it is characterized by erythematous lesions with variable intensity and extension, often affecting the palatal mucosa of denture wearers. The treatment of choice consists of topical antifungals, such as nystatin or miconazole and proper denture hygiene, as well as removal of the denture at night [ 3 ]. However, there are cases of therapeutic failure and of rapid recurrence after treatment, especially in the absence of proper denture hygiene [ 3 ]. Moreover, the increase in resistance of *Candida* spp. yeasts to commercially available antifungals in recent years, as well as their toxicity and the risk of drug interactions [ 4 ], have restricted their use by the elderly [ 5 ]. Natural products, many of which do not have such disadvantages, have attracted interest as an alternative to synthetic medications [ 6 ]. Among these natural products, propolis has demonstrated anti-inflammatory [ 7 ], anti-ulcerative [ 8 ], antimicrobial [ 9 ], healing [ 10 ] and antioxidant [ 11 ] properties, as well as fungicidal effects against various species of *Candida*, including *C. albicans*, *C. parapsilosis* and *C. glabrata*, in *In vitro* studies [ 7 , 12 - 13 ]. Various studies have demonstrated the efficacy of propolis for the treatment of DS [ 14 - 17 ]. Propolis has many components, and its composition may vary depending on the production region, soil type, bee subspecies, season of the year, harvesting techniques and product standardization, among other factors, which could affect the chemical profile and the biological properties of extracts [ 18 ]. Consequently, its clinical properties may vary, which could explain the different results obtained by some researchers [ 15 - 17 ]. Pomegranate (*Punica granatum*: Lythraceae) is also known for its medicinal properties and has aroused the interest of researchers. Pomegranate is traditionally indicated as an anti-inflammatory and oral antiseptic agent [ 19 ]. *In vitro* studies have demonstrated it can inhibit *C. albicans* [ 20 ], in addition to antimicrobial activity against cariogenic bacteria [ 21 ] and antioxidant properties [ 22 ]. Punicalagin, an ellagitannin found in pomegranate, is one of its main antimicrobial constituents [ 22 ]; it also has antifungal activity against *C. albicans* and *C. parapsilosis*. [ 23 ] While the specific mechanisms of action of tannins against *Candida* spp. are still unclear [ 20 ], *In vitro* activity against *Candida* spp. [ 23 - 25 ] and removal of 90% of the biofilm formed on the surface of the acrylic resin of removable dentures has been demonstrated in *In vitro* tests with pomegranate extract added to denture adhesives [ 25 ]. Though various *In vitro* studies have been made on the effect of pomegranate extract on oral pathogens [ 20 - 21 , 24 - 25 ], only one clinical trial has tested its efficacy for the treatment of DS [ 26 ].

An experimental study demonstrated that an ethanolic extract of pomegranate peel (pericarp) caused morphological and structural changes in *Candida* species, detected by transmission electron microscopy, producing irregularities in membranes and hyphae, thinner cell walls and cytoplasmic vacuolization [ 24 ], demonstrating the potential antimicrobial activity of *P. granatum*. Another study showed concentration-dependent activity against *Candida* spp. of an alcoholic extract of *P. granatum* peel in salivary samples collected from patients with oral candidiasis [ 25 ]. A clinical trial revealed clinical and microbiological efficacy of a laboratory-produced *P. granatum* gel in the treatment of patients with DS [ 25 ].

Pomegranate and propolis extracts have not been tested together for DS treatment. A propolis-pomegranate buccal spray product (Apiromã®) is commercially available in Brazil; it consists of a standardized propolis extract (EPP-AF®), composed mainly of Brazilian green propolis [ 10 ] at a concentration of 0.5%, and 0.9% pomegranate extract. This product is normally used as an oral antiseptic for the treatment of upper airway infections. Given the possibility of synergy of propolis and pomegranate, we decided to assess the efficacy of pomegranate/propolis spray for the treatment of DS.

## Materials and Method

### Study design

This was an open-label, parallel two-arm, non-inferiority randomized clinical trial with a miconazole control. The study was approved by the Human Research Ethics Committee of the School of Health Sciences at the University of Brasília (process 3.033.121; Certificate of Presentations of Ethical Appreciation number 81889717. 7.0000.0030) and it was registered at the Brazilian Registry of Clinical Trials under code RBR-6YF4CV. Informed consent was obtained from all participants included in this study.

### Participants

Participants were recruited at the Ceilândia Unit of the Social Service for Trade of the Federal District between June and August 2018. Inclusion criteria were age ≥60 years, wearer of removable partial or complete denture with palatal coverage, and diagnosis of DS. Exclusion criteria were treatments with any kind of antifungal product, antibiotic or anti-inflammatory drug for two months prior to recruitment; dementia or cognitive deficit were also included as exclusion criteria. Demographic and clinical history data were collected via anamnesis at day 0 (T0) of the study. The participants answered questions about denture age, frequency of daily oral hygiene, and habit of wearing the denture while sleeping. DS was diagnosed via intra-oral examination by a trained dental surgeon at T0, T7 and T14. Newton’s classification [ 27 ] was used for the lesions, categorized as I (localized inflammation or petechiae), II (diffuse erythema involving all or part of the hard palate covered by the denture), and III (erythema associated with papillary hyperplasia in the area covered by the denture). All oral lesions were photographed and reviewed by a second blinded evaluator. In case of disagreement between the evaluators, the photos were reviewed and a reclassification was made. 

### Randomization and allocation

Participants were randomly assigned to study groups according to a random number table produced with http://www.graphpad.com/quickcalcs, GraphPad Software, Inc. A second researcher generated the random allocation sequence, and the principal researcher allocated the participants into the groups (MIC- miconazole group; PP– propolis-pomegranate group) based on this random sequence. 

### Interventions

Participants applied 2% miconazole oral gel (Daktarin® Gel Oral, Janssen-Cilag Pharmaceutics) or a water-based spray containing 0.5% propolis extract and 0.9% P. granatum extract (Apiromã®, Apis Flora Industrial & Comercial Ltda, Ribeirão Preto, Brazil) to their dentures. Apiromã® contains standardized propolis extract (EPP-AF®, Apis Flora Industrial & Comercial Ltda, Ribeirão Preto, Brazil), *P. granatum* extract, honey, xanthan gum, and water. The EPP-AF® propolis extract is obtained from a blend of propolis from several Brazilian regions, though it mainly consists of green propolis originating from Baccharis dracunculifolia [ 10 ]. Participants were instructed to perform prosthesis hygiene by brushing with dentifrice and to sprinkle 0.12% chlorhexidine digluconate solution on the inner surface of the denture to remove excess chlorhexidine, and to apply a thin layer of 2% miconazole oral gel or to spray Apiromã® on this surface, three times a day for 14 days. All participants received instructions on oral hygiene, denture hygiene maintenance, use of the products, and removal of dentures for nighttime sleep. The dentures were stored at night in water. Participants received a tube of miconazole or Apiromã® buccal spray with preweighed amounts, 0.12% chlorhexidine solution, and written instructions on the use of the products. 

For microbial load analysis, participants rinsed their mouths with 20 mL of sterile 0.9% sodium chloride at T0 and T14 in the dental clinic. The rinse samples were collected into Falcon tubes, kept on ice, and processed up to one hour after collection. They were centrifuged for 15 minutes at 25°C at 3,000xg; the supernatant was discarded, and the pellets were resuspended in 1mL of 0.9% sodium chloride solution, and then the suspensions were diluted 10:1 successively, to provide 10-1 to 10-4 dilutions. Fifteen microliters of each dilution were seeded onto sterile Petri plates containing Sabouraud dextrose agar supplemented with chloramphenicol (0.5g/L) (Difco, Detroit, MI, USA), using the hanging drop culture method. Another plate, with CHROMagar *Candida*® supplemented with chloramphenicol (0.5g/L) (CHROMagar Company, Paris, France) was streaked with 50 µL of the undiluted suspension for identification of the *Candida* spp. colonies. The Petri plates were incubated at 37°C for 48–72 h. The colonies were identified on CHROMagar *Candida*® medium, and the fungal load was counted on the Sabouraud dextrose agar supplemented with chloramphenicol medium and expressed in colony-forming units per milliliter of oral lavage solution (CFU/mL). The miconazole tubes and Apiromã® flasks were weighed at T0, T7 and at the end of the study (T14) for assessment of treatment compliance. The patients who used the product within the mean consumption amount, calculated by average daily frequency of product use, within two standard deviations, were considered compliant with treatment. Adverse events reported by the participants were recorded. 

A seven-point scale adapted from Moskowitz *et al.* [ 28 ] was used to evaluate product acceptance. The seven-point hedonic scale is a balanced bipolar scale around neutral at the center with three positive and three negative categories on each side to provide a single continuum of degree of like and dislike. Patients were asked to rate the products using: 1= ‘Strongly disliked’; 2= ‘Moderately disliked’; 3= ‘Slightly disliked’; 4= ‘Indifferent’; 5= ‘Slightly liked’; 6= ’Moderately liked’ and 7= ‘Strongly liked’.

### Outcomes

Clinical cure (rate of DS resolution), defined as absence of lesions, was the main outcome. Secondary outcomes were fungal load reduction (greater than 90% reduction of CFU/mL counts), rate of adverse events, and product acceptance.

### Sample size

Sample size was estimated as described by Chow *et al.* [ 29 ] for parallel two-arm studies with dichotomous outcomes. Based on previous data [ 14 ], a non-inferiority margin of 0.15 (15% variation between treatments is considered clinically nonsignificant), 70% response rate for miconazole, 70% response rate for Apiromã®, 5% significance level, and 80% power of the study were adopted. Accordingly, a sample size of 18 individuals per arm was estimated. Given an estimated 10% loss to follow-up, 20 participants per arm were recruited.

### Statistical analysis

The data were reported as means or medians, with the respective measures of variability. The Mann-Whitney test was used to analyze the magnitude of the effect (clinical efficacy) and product acceptance. A non-inferiority interval was calculated from the data observed in the standard treatment group, with a non-inferiority margin of 15%. The chi-square test was used for comparison of cure rates and rates of adverse events. GraphPad Prism v 7.0 was used for statistical analyses. *p* Values <0.05 were considered statistically significant. 

## Results

**Figure 1:**
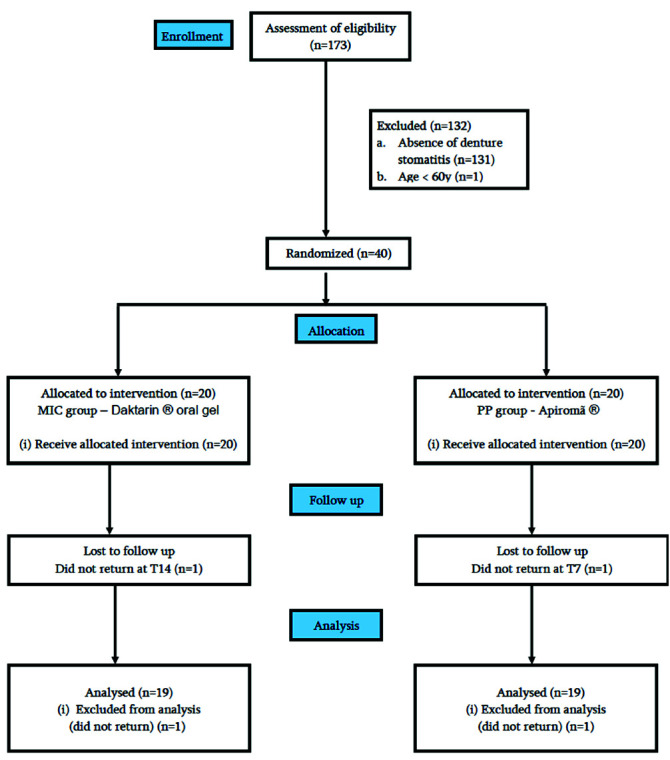
CONSORT flowchart diagram of the study from enrollment of the participants until analysis

A total of 172 participants were assessed between June and August 2018. Out of 172 participants, 132 were excluded (131 did not have DS and one was younger than 60 years old). Consequently, 40 individuals were included in the study. Among the 40 participants, one from each group was lost to follow-up. The Consolidated Standards of Reporting Trials flowchart is shown in Figure 1.

Table 1 summarizes the characteristics of the groups and initial classification of DS lesions.

**Table 1:** Characteristics of the treatment groups and initial classification of denture stomatitis. The dentures were treated with propolis-pomegranate spray (PP) or miconazole gel (MIC). Data expressed as mean ± SD or median and interquartile range 25 to 75%. a Mann Whitney test, b Chi-square test

	PP group	MIC group	*p* Value
Age (years)	72(±6)	69(±5)	0.02^a^
Skin color* (% were white)	65	50	0.52^b^
Time of denture use (years)	30(±6)	28(±13)	0.62^a^
Age of current denture(years)	9(±7.6)	7.7(±5.6)	0.12^a^
Frequency of denture hygiene (times/day)	3(2-3)	3(1-3)	0.23^b^
Newton’ score (T0)**	1.8(±0.8)	1.9(±0.8)	0.70^a^

**Figure 2:**
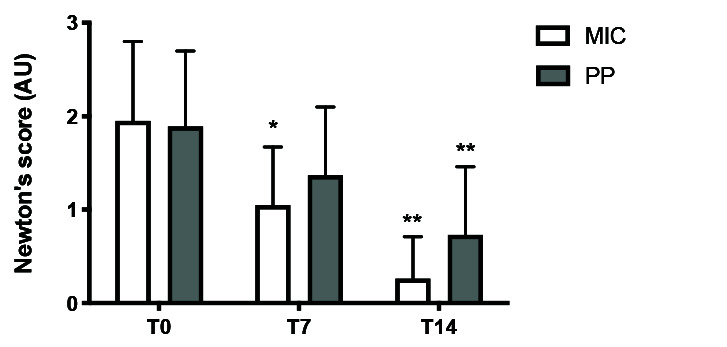
Newton's score at T0, T7 and T14 for the miconazole (MIC) (white bars) and propolis-pomegranate spray (PP) groups (gray bars) * *p*< 0.05, ** *p*< 0.001; T7 or T14 vs. T0

Figure 2 shows a reduction in Newton’s scores in the first and second weeks of treatment. After the first week, there was clinical improvement only in the MIC group. After the second week, there was clinical improvement in both groups. The effect magnitude (given by the difference in Newton’s scores at T0 and T14) was 1.6 points for the MIC group and 1.2 points for the PP group. At the end of the study, the mean score for the MIC group was 0.3. The PP group had a mean score of 0.7 at T14. The effect magnitude was greater for the MIC group (p= 0.02).

**Figure 3:**
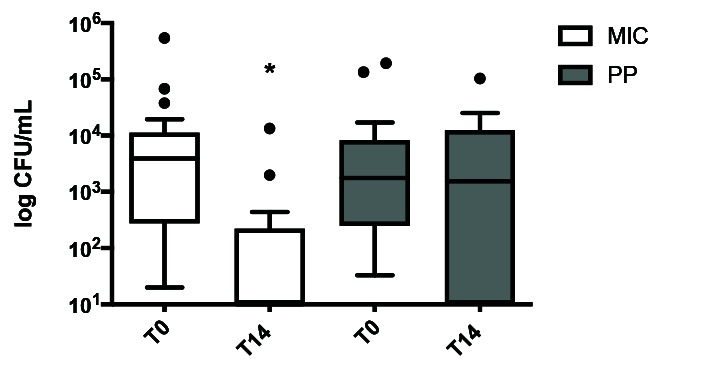
Tukey’s box plot of fungal load (log CFU/mL in 20 ml buccal wash) at T0 and T14 for the miconazole (MIC) (white bars) and propolis-pomegranate (PP) (gray bars) groups. There was a significant reduction in CFU/mL only in the MIC group (*p< 0.0001 at T0 vs. T14)

The clinical cure rate was 75% for the MIC group and 40% for the PP group (Table 2); consequently, the hypothesis of non-inferiority of Apiromã® compared to miconazole was rejected. Also, CFU/mL in 20ml buccal wash decreased in the MIC group (p< 0.0001), but not in the PP group (p= 0.70) (Figure 3). 

**Table 2:** Cure ratea of dental stomatitis (DS) (%) based on the non-inferiority margin (NI) for Daktarin® gel oral and Apiromã®

Product	Cure rate (%)	NI (15%)
Daktarin® gel oral	75.0	52.0 – 98.0
Apiromã ®	40.0	16.0 – 64.0

According to the hedonic scale data, product acceptance was similar in the two groups (p= 0.72). The median was 5 (4.2-6.0) for the MIC group and 5.5 (4.2-6.0) for the PP group (Table 3).

**Table 3:** Distribution of *Candida* spp. between miconazole (MIC) and propolis-pomegranate (PP) groups – Absolute and relative (%) numbers of patients colonized by specific *Candida* spp. Some patients had more than one species of *Candida*

	MIC group		PP group	
T0^a^	T14^b^	T0^a^	T14^b^
*C. albicans*	10(50%)	5(26.3%)	15(75%)	16 (84.16%)
*C. tropicalis*	12(60%)	2(10.52%)	11(55%)	6 (31.56%)
*C. krusei*	5(25%)	1(5.26%)	7(35%)	3 (15.78%)
*C. kefyr*	1(5%)	0	0	1 (5.26%)
*C. glabrata*	1(5%)	0	2(10%)	3 (15.78%)
Other species	1(5%)	1(5.26%)	0	1 (5.26%)

## Discussion

An evaluation was made of a commercial formula (Apiromã®) that has a completely natural product composition, which includes propolis (0.5%), pomegranate extract (0.9%), honey and water, without any chemical additives or preservatives, in a vaporizer presentation (buccal spray), compared with 2% miconazole gel (Daktarin®). Fortuitously, besides anti-*Candida* effects, propolis and pomegranate have anti-inflammatory and antioxidant properties [ 10 , 22 ], not directly measured in this study, which potentially could provide additional benefits for the treatment of DS. The Apiromã® product label recommends the application of 3-4 sprays/use, when necessary. It is available in pharmacies in Brazil as a mouth-refreshing product. This recommendation of 3-4 sprays is equivalent to 1.5-2.0 mg of propolis and 2.7-3.6mg of pomegranate extract per application, based on our lab analyses. It was assumed that the efficacy of Apiromã® would be non-inferior to that of 2% miconazole gel for the treatment of DS. The non-inferiority margin (15%) was adopted for analysis of the treatment effect, considered as clinical cure. Given the higher non-inferiority margin of Apiromã® (0.64) compared to that obtained for miconazole (0.52-0.98), the efficacy of this product was significantly lower than that of the control miconazole treatment. In addition, from a clinical standpoint, the propolis-pomegranate spray product did not have good efficacy, since the clinical cure rate was low (40%).

This product did not reduce the fungal load significantly, demonstrating a lack of antifungal efficacy. The clinical trial performed by Pina *et al.* [ 14 ] in which they used a mucoadhesive gel containing the same propolis extract used in this study (EPP-AF®), but at a higher concentration (2%), had a higher clinical cure rate (70%) for DS, but also did not have antifungal efficacy. These findings could be explained by the effect of propolis on *C. albicans* dimorphism, provoking the transition of more pathogenic forms, these being pseudohyphae and hyphae to yeasts, by affecting the immune system [ 30 ]. This phenotypic change from hyphae and pseudohyphae to yeasts is induced by propolis without quantitative reduction in CFU/mL [ 13 ]. These findings lead to the conclusions that propolis does not have an antifungal effect at the tested doses (1.5–2.0mg of propolis per application of the buccal spray), and that its anti-inflammatory and healing properties predominate over its antifungal activity [ 14 ]. 

Another study demonstrated clinical and microbiological efficacy of green propolis when used as 2.5% gel and 24% mouth rinse, applied four times a day for seven days for DS [ 15 ], a dosage much higher than that used in our current study (0.5%), indicating that the posology should be adjusted.

The vehicle in which the product is applied could also affect results. Propolis in a mucoadhesive formulation would remain in contact with the mucosa for a longer period compared to the application of a liquid propolis solution to the denture surface. A liquid formulation was compared with a gel, which have completely different durations of effective contact with the treatment area, affecting permeation of the active ingredients. 

The two treatment groups had similar product acceptance. Two participants of the PP group reported a burning sensation and one described having felt a very sweet taste; but they did not discontinue the treatment with Apiromã®. No important adverse events were observed for any of the products, demonstrating their safety. The potential advantages of a product, containing pomegranate blended with propolis are lower cost, fewer drug interactions, fewer adverse effects, and lower potential for fungal resistance. In addition to being a natural and edible product, propolis does not have any risks of drug interactions [ 31 ], reducing the risk of adverse reactions when compared to industrialized products. Toxicological studies with humans have shown that pomegranate is safe for obese and diabetic individuals [ 17 ]. Of note, some (40%) of the patients had clinical improvement after the use of Apiromã®, which encourages the future development of a mucoadhesive formulation with higher concentrations of propolis and pomegranate, and/or changes in the posology, increasing the amount of propolis and pomegranate.

The positive points of this study included successful use of an easily available commercial product made with standardized propolis extract (EPP-AF®) and the study design (randomized controlled clinical trial that assessed patients with DS and an efficient control). The impossibility of conducting a blinded study because of the characteristic aspect and smell of propolis, summed with the fact that the dosage used was lower than the requested by the package were limitations of this study. 

## Conclusion

This randomized controlled trial demonstrated that the 0.5% propolis and 0.9% pomegranate extract buccal spray product Apiromã® can help control stomatitis in denture users, though it is less efficient than 2% miconazole gel, which is commonly recommended and used. 

## Acknowledgement

This study was partially financed by the Conselho Nacional de Desenvolvimento Científico e Tecnológico (C-NPq), grants 205555/2018-7 and 433790/2018-0 to VPM; by the Fundação de Amparo à Pesquisa do Distrito Federal (FAPDF), grant 00193.00000100/2019-6 to E-NL; and by the Coordenação de Aperfeiçoamento de Pessoal de Nível Superior (CAPES) through a PhD scholarship for HSM.

## Funding Statement 

Apis Flora Comercial & Industrial Ltda. donated the Daktarin Gel Oral® and Apiromã®. Research funded by the Brazilian research funding organization Fundação de Amparo à Pesquisa do Estado de São Paulo (FAP-ESP).

## Data Availability

All data of this study are available from the corresponding author upon reasonable request.

## Conflict of Interest

Andresa Aparecida Berretta and Jessica Aparecida de Lima are employed at Apis Flora Industrial e Comercial Ltda., Ribeirão Preto, SP, Brazil. The other authors confirm that they have no known conflicts of interest associated with this publication.
